# Obstructive sleep apnea phenotypes eligible for pharmacological treatment

**DOI:** 10.3389/frsle.2023.1261276

**Published:** 2023-09-01

**Authors:** Marie Bruyneel

**Affiliations:** ^1^Department of Pneumology, Centre Hospitalier Universitaire (CHU) Saint-Pierre, Brussels, Belgium; ^2^Department of Pneumology, Centre Hospitalier Universitaire (CHU) Brugmann, Brussels, Belgium; ^3^Department of Pneumology, Université Libre de Bruxelles, Brussels, Belgium

**Keywords:** obstructive sleep apnea, phenotype, endotype, PALM classification, treatable traits, reboxetine, oxybutinine, solriamfetol

## Abstract

Obstructive sleep apnea (OSA) is a common disorder. Its prevalence is increasing worldwide, partially due to increasing rates of obesity, and OSA has a well-documented impact on physical health (increased risk of cardiovascular and metabolic disorders) and mental health, as well as major socioeconomic implications. Although continuous positive airway pressure treatment (CPAP) remains the primary therapeutic intervention for moderate to severe OSA, other treatment strategies such as weight loss, positional therapy, mandibular advancement devices (MAD), surgical treatment, myofunctional therapy of upper airways (UA) muscles and hypoglossal nerve stimulation are increasingly used. Recently, several trials have demonstrated the clinical potential for various pharmacological treatments that aim to improve UA muscle dysfunction, loop gain, or excessive daytime sleepiness. In line with the highly heterogeneous clinical picture of OSA, recent identification of different clinical phenotypes has been documented. Comorbidities, incident cardiovascular risk, and response to CPAP may vary significantly among phenotypes. With this in mind, the purpose of this review is to summarize the data on OSA phenotypes that may respond to pharmacological approaches.

## 1. Introduction

Obstructive sleep apnea (OSA) is a common medical disorder that is due to total or partial pharyngeal collapse and temporary upper airway (UA) obstruction during sleep, resulting in recurrent episodes of apnea or hypopnea. Due to increasing rates of obesity and to the aging of the population, the prevalence of OSA is increasing, affecting about 30% of men and 13% of women in Europe (Heinzer et al., 2015). Factors associated with OSA, such as intermittent hypoxia (IH), oxidative stress, systemic inflammation, sympathetic activation, respiratory efforts, and sleep fragmentation can provoke cardiometabolic conditions. In particular, OSA is an independent risk factor for cardiovascular disorders, such as hypertension (HT), arrhythmia, coronary heart disease, and stroke (Marin et al., 2005; Mehra et al., 2009; Arzt et al., 2017), and some metabolic disorders (e.g., diabetes mellitus, disorders of lipid metabolism) have also been shown to be associated with OSA (Tamura et al., 2008; Lam et al., 2010). The mainstay of OSA treatment is continuous positive airway pressure (CPAP), but numerous other treatments have also been demonstrated to be effective in well-selected patients such as mandibular advancement devices (MAD), positional therapy (PT), UA and maxilla-mandibular surgery (Gottlieb and Punjabi, 2020; Gambino et al., 2022), myofunctional therapy of UA muscles (Carrasco-Llatas et al., 2021), electrical stimulation and hypoglossal nerve stimulation.

Diagnosis of OSAS (OSA syndrome) is based on criteria that indicate the presence of obstructive respiratory events through measurement of a combination of symptoms, comorbidities, and poly(somno)graphic [P(S)G] recordings (American Academy of Sleep Medicine, 2014). The preferred reference diagnostic method is in-lab PSG (Gambino et al., 2022), but home sleep testing (PG) can also be used for OSA diagnosis in patients who have a high probability of moderate-to-severe OSA based on pre-test evaluation (American Academy of Sleep Medicine, 2014).

In order to express the severity of OSA, obstructive AHI values of 5–14, 15–29, and >30 are used to define mild, moderate, and severe OSA, respectively. However, AHI is poorly correlated with the severity of clinical symptoms (Pevernagie et al., 2020), despite the fact that there is a linear relationship between AHI and HT (Peppard et al., 2000) and AHI correlates with overall mortality in OSA patients (Kendzerska et al., 2014). The use of AHI metrics is now controversial, given the limitations associated with them (first-night effect, night-to-night variability, over- or under-estimation of events depending on the sensors used and definitions of hypopnea). Hypoxemia appears to better reflect the impact of OSA on the occurrence of cardiovascular and metabolic comorbidities. A recent systematic review concludes that oxygen desaturation index (ODI) (value of 4%) ≥15 events/h should be considered as the cut-off for diagnosing OSA with a specificity from 75 to 98% and positive predictive value of 97% (Rashid et al., 2021).

There is also significant heterogeneity in the clinical picture of OSA. In the last decade, different clinical phenotypes have been identified, highlighting clusters of OSA with different symptomologies and comorbidities, despite similar AHI on PSG (Zinchuk and Yaggi, 2020). Indeed, pathophysiological processes generated by obstructive events can vary among patients (e.g., IH, sympathetic activation, systemic inflammation, oxidative stress, sleep fragmentation, and respiratory efforts) and can lead to a wide range of symptoms and comorbidities.

**Figure 1:**
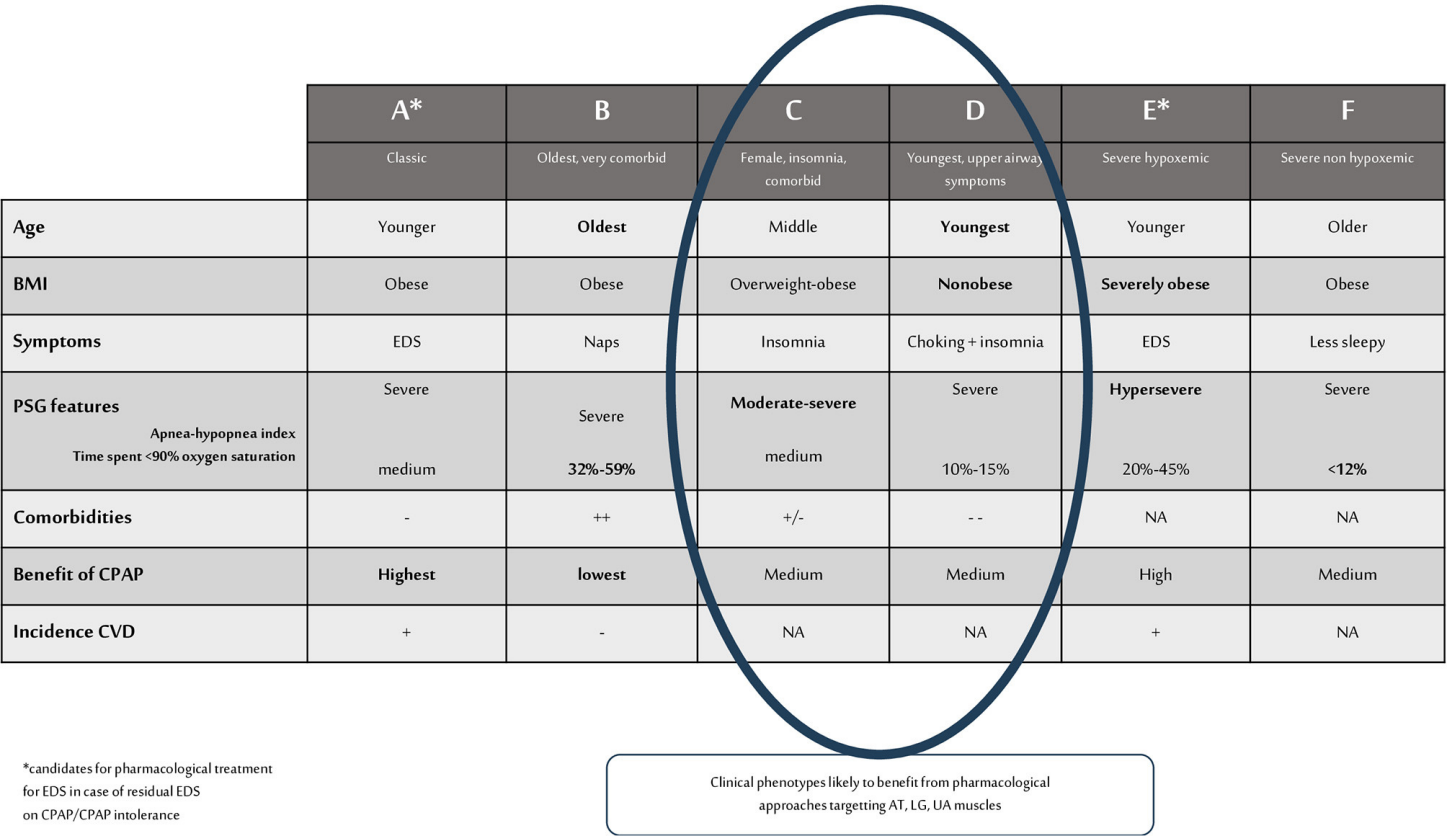
Summary of OSA clinical phenotypes and phenotypes likely to benefit from pharmacological approaches that target AT, LG, UA muscles. BMI, body mass index; PSG, polysomnographic; CPAP, continuous positive airway pressure; EDS, excessive daytime sleepiness; NA, not assessed; CVD, cardiovascular disease; AT, arousal threshold; LG, loop gain; UA, upper airways.

An OSA phenotype thus refers to a category of patients with OSA that can be distinguished from others by a single disease feature, or a combination of disease features, in relation to clinically meaningful attributes such as symptoms, P(S)G characteristics (hypoxemia, AHI), response to therapy, comorbidities, and incident cardiovascular disorders (Zinchuk et al., 2017). A summary of OSA clinical phenotypes is illustrated in Figure 1. In addition, underlying pathophysiological traits leading to these different clinical phenotypes are described as “endotypes” and can be of importance for guiding response to therapy (Light et al., 2019). Anatomical factor is undoubtedly the main cause of OSA. Narrowed and/or collapsible UA during sleep are responsible for the occurrence of respiratory events in the majority of patients, which explains why most existing therapies for OSA are geared toward reversing the anatomical problem (e.g., CPAP, MAD, UA surgery,…). However, in addition to impaired UA anatomy, other factors contribute to the different endotypes of OSA (Eckert, 2018).

The PALM classification is typically used to describe OSA endotypes based on dynamic factors that have been implicated in OSA occurrence. P stands for critical pressure, A for arousal threshold, L for loop gain, and M for muscle recovery (Bosi et al., 2020). The “P” of the PALM classification refers to the anatomic collapsibility reflected by the critical occlusion pressure (Pcrit). Deposition of fat in the UA and abdomen (reduced lung volume) increases anatomic collapsibility. Snoring and repeated pharyngeal depressions during obstructive events lead to chronic inflammation of the soft tissue, reducing the diameter of the UA and increasing anatomic collapsibility. Collapsibility is also increased in the supine position with rising of the diaphragm, and backdrop of the jaw and tongue. Pcrit is not routinely measured but can be assessed during a CPAP trial and PSG, by reducing the CPAP levels with the aim of determining the pressure at which the UA occludes.

The “A” in the PALM classification is the arousal threshold (AT) which is defined as the level of inspiratory effort at which obstructive events terminate with an arousal from sleep. If all of the mechanisms successfully achieve UA patency and sustainable ventilation, an arousal from sleep may not be required to support ventilation. The threshold required to achieve UA reopening (associated with sustainable ventilation) is defined as the threshold of effective recruitment (Ter). The arousal at the end of an obstructive event occurs when the AT <Ter or when hyperventilation follows UA reopening (stimulation of arousal center). A low AT causes sleep fragmentation, and ventilatory and pharyngeal muscular instability, promoting obstructive event recurrence and excessive daytime sleepiness (EDS).

The “L” in the PALM classification refers to the loop gain (LG), or ventilatory control, and includes three components including the control component, via chemoreceptors, the exchange component, via the lungs, and the connection component, via the blood circulation. LG is performed by two mechanisms: control gain and plant gain. The first refers to the degree of response of the respiratory system to the change in PaCO_2_. Plant gain refers to the ability of the respiratory system to respond to a reduction in CO_2_ by ventilation. LG > 1 (high control gain) is related to a (hyper-)sensitive system, leading to excessive ventilatory response (periodic breathing), and LG <1 is associated with a more stable ventilatory system. Currently, the reference methods for assessing LG require an overnight stay in a specialized physiology laboratory with hypoxic/hypercapnic gas administration or CPAP manipulation. Messineo et al. (2018) proposed the breath-holding maneuver as a cost-effective and easy daytime test to identify high LG in OSA patients.

The “M” in the PALM classification refers to muscular UA gain. Several muscles are responsible for UA patency including the muscles that regulate the position of the hyoid bone (geniohyoid and sternohyoid), muscles of the base of the tongue (mainly the genioglossus), pharyngeal constrictors, and muscles of the soft palate (tensor and elevator palatini). The genioglossus is the main dilator muscle of the UA. Its most important functions are tongue depression and protrusion. The muscle is innervated by the medial branch of the hypoglossal nerve, decreasing its activity during expiration and increasing it during inspiration. Increasing the activity of this muscle and preventing hypotonia during sleep are OSA treatment targets, as is the recently developed hypoglossal nerve stimulation. Conventional electromyography (EMG), performed with needles, is the traditional method for identifying hypotonic patients, but it is invasive and impractical for routine clinical use. Transmembraneous EMG, the Iowa Oral Performance Instrument (IOPI), the Tongue digital spoon (TDS), OMES (Orofacial Myofunctional Evaluation With Scores) and the extended OMES protocol are simple, affordable, non-invasive methods that allow repeated measurements over time to identify “M” patients (O'Connor-Reina et al., 2023).

Approximately 20% of OSA patients have high anatomic collapsibility and 80% have an association between anatomic disharmonies and anomalies in AT/muscle responsiveness/LG (Eckert, 2018).

PALM 1 (23%) represents very high UA collapsibility (Pcrit > 2.5 cm H_2_O). In this case, weight loss, PT, MAD, CPAP, and UA surgery are the first-line treatments (anatomical treatment).

PALM 2 (57%) represents an intermediate collapsibility (Pcrit between +2.5 and −2.5 cm H_2_O). The patients of subgroup 2a (no major non-anatomical impairment) are candidates for anatomical treatment, see PALM 1. The patients of subgroup 2b (one or more non-anatomical impairments) are candidates for a combination of anatomical and non-anatomical treatments (e.g., drugs, hypoglossal nerve stimulation).

PALM 3 (19%) represents a low UA collapsibility (Pcrit <−2.5cm H_2_O) with a therapeutic level of CPAP ≤ 8 cm H_2_O.The treatment for these patients includes non-CPAP treatment options: weight loss, MAD, oxygen, and drugs targeting the LG or the AT.

To summarize, we can expect that PALM 2b and three patients, suffering mainly from non-anatomical impairments, are likely to respond to treatments that lead to an increase in UA muscle activity or AT, or to a decrease in LG (Eckert, 2018). These treatments can be offered by pharmacological approaches. Other targets for pharmacological treatment are symptoms, particularly EDS. This is the main symptom of OSAS and can be difficult to alleviate despite an adequate anatomical or non-anatomical specific OSA treatment.

## 2. Pharmacological approaches in OSA

**Figure 2:**
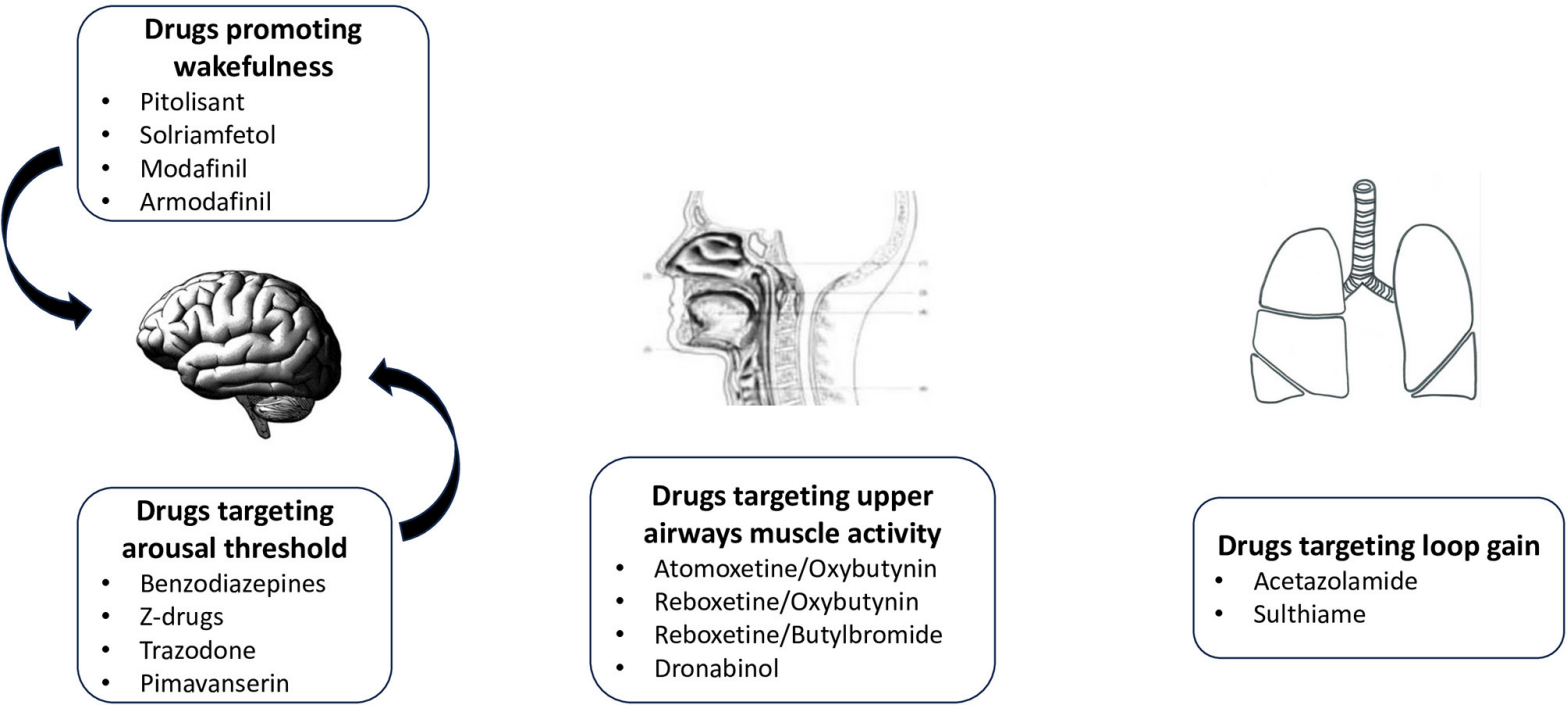
Targets of pharmacological treatments in OSA.

Pharmacological attempt to treat OSA is not recent. In 2019, Gaisl et al. (2019) performed a systematic review and network meta-analysis on the topic, including 44 drugs studied in 58 RCT. Results were very disappointing since most trials were not adequately powered. Only acetazolamide was shown to reduced AHI significantly. Authors summarized very nicely all the potential targets for experimental pharmacological treatments in OSA. They also emphasized that pharmacological treatments were mostly “add-on” rather than “stand-alone” treatments as the largest reduction in AHI was observed in mild to moderate OSA. Since this analysis, new positive studies have been published, including more severe patients, providing a better understanding of effective therapeutic targets. The diverse types of drugs, acting on different pathophysiological targets, are summarized in Figure 2.

### 2.1. Targeting loop gain

To counter hypersensitive ventilatory control, acetazolamide, a carbonic anhydrase inhibitor that produces metabolic acidosis leading to increased baseline ventilation, has been studied in OSA (Edwards et al., 2012). Thirteen randomized controlled trials (RCTs) were recently pooled in a meta-analysis and systematic review that concluded that the drug was associated with an AHI decrease of 14/h and greater efficacy at higher dosages, up to 500 mg/day (Schmickl et al., 2020). Study durations were generally very short, with a median of 6 days, and efficacy was similar for central and obstructive sleep apnea disorders.

More recently, the carbonic anhydrase inhibitor, sulthiame, has been studied in an RCT conducted in 68 patients with moderate and/or severe OSA who could not tolerate CPAP treatment (Hedner et al., 2022). The drug, administered for 4 weeks, reduced AHI from 55.2 to 33.0 events/h (−41.0%) in the 400-mg group and from 61.1 to 40.6 events/h (−32.1%) in the 200-mg group. Sulthiame reduced AHI by more than 20 events/h, one of the strongest reductions achieved in a drug trial in OSA (Hedner et al., 2022).

### 2.2. Targeting arousal threshold

During obstructive respiratory events, the amount of respiratory effort can vary greatly between individuals and according to sleep stage. Respiratory event-related arousals induce breathing instability, such that a low AT has been shown to be an important predictor of OSA severity (Dutta et al., 2021). Hypnotics (benzodiazepines and Z-drugs) usually target the GABAergic system (GABA-A receptor agonists) and depress the central nervous system (CNS), promoting sleep. Initial studies with benzodiazepines reported an increase in hypoxemia and respiratory event duration in OSA, related to CNS depression and myorelaxation, but these studies were performed in very severe OSA with marked nocturnal hypoxemia (Berry et al., 1995). Since these observations, numerous publications have been written on the subject, highlighting a decrease in AHI in some OSA patients, especially those without severe hypoxemia and with low to moderate AT (Carter and Eckert, 2021). In one RCT on 21 patients, 10 mg zolpidem was found to increase AT, but also to promote a positive effect on OSA by stimulating genioglossus activity during sleep (Carberry et al., 2017). However, the sample size in the Z-drugs studies was very small and the drugs were used generally for only 1–2 nights. Contradictory results were obtained with trazodone, a tricyclic anti-depressant with hypnotic properties (Carter and Eckert, 2021).

Recently, an RCT was conducted with pimavanserin in 18 OSA patients. Pimavanserin is an anti-serotoninergic capable of suppressing CO_2_-mediated arousals without affecting the respiratory motor response in animal models. Results were disappointing for the group as a whole, but a subset of patients exhibited an increase in AT and a small decrease in OSA severity (Messineo et al., 2022).

### 2.3. Targeting upper airway dilating muscles

At the level of the UA muscles, two important phenomena contribute to UA collapse: sleep-related withdrawal of endogenous noradrenergic drive is a major cause of genioglossus hypotonia during non-rapid eye movement (NREM) sleep and, in REM sleep, active muscarinic inhibition induces pharyngeal hypotonia.

Initial studies with noradrenergic agents (e.g., desipramine, proptyline) led to disappointing results. An RCT designed to assess the effect of a selective norepinephrine reuptake inhibitor (atomoxetine) administered in combination with an antimuscarinic drug (oxybutynin) on OSA severity and genioglossus responsiveness was performed for one night in 20 patients, and provided a reduction in AHI of 63% (Taranto-Montemurro et al., 2019). Interestingly, nine patients received the drugs separately, and they were ineffective alone. More recently, the same study group tried the combination of reboxetine (a noradrenergic agent) and oxybutynin for 1 week in a group of 16 severe OSA patients (Perger et al., 2022). AHI was reduced by 59%, and the treatment combination was also effective on hypoxemia and oxygen desaturation index (ODI).

The combination of reboxetine and the anti-muscarinic agent hyoscine butylbromide was also tried in an RCT that included 12 patients, and demonstrated an interesting reduction in AHI, from 51 to 33/h, related to increased UA patency via muscle activation (Lim et al., 2021).

### 2.4. Other targets

One RCT has explored two doses of dronabinol (2.5 and 10 mg), a cannabinoid Type 1 and 2 receptor agonist, on OSA (Carley et al., 2018). The mechanism of action of these drugs on respiratory pattern and UA stability remains unknown but seems to be related to increased vagal afferent activity. Afferent vagal neurons express a range of somatic receptors, including inhibitory cannabinoid Type 1 receptors and excitatory 5-HT_3_ receptors. Activation of cannabinoid Type 1 receptors would be expected to attenuate apnea expression, while activation of nodose ganglion 5-HT_3_ receptors is likely to increase apnea propensity. In this series of 73 moderate OSA patients, dronabinol, taken for 42 days, achieved a significant reduction in AHI (32%) and EDS, opening the door to further trials (Carley et al., 2018).

## 3. Alternative approaches: symptom suppression

EDS is a very common symptom in OSA that can persist despite CPAP in 6–15% of patients (Pépin et al., 2021). Pitolisant is a selective histamine H3 receptor antagonist with strong wake-promoting effects. Pépin et al. (2021) performed a large RCT for 12 weeks in 244 OSA patients currently on CPAP, with increasing doses of pitolisant, and reported a significant reduction in EDS, measured by the Epworth Sleepiness Scale (ESS), from 15 to 9. Pitolisant was also studied in OSA patients who refused or did not tolerate CPAP (*n* = 268), with similar results and a very good safety profile (Dauvilliers et al., 2020).

Solriamfetol, a dual dopamine-norepinephrine reuptake inhibitor, was studied at different dosages in three RCTs for persistent EDS in OSA patients, with positive impacts on ESS and a good safety profile (Wang et al., 2021).

Finally, modafinil and armodafinil, both dopamine reuptake inhibitors, were extensively studied in RCTs for residual EDS in CPAP users, or in sleepy OSA patients who refused or did not tolerate CPAP with a significant decrease in ESS and a good safety profile (Kuan et al., 2016). However, these medications tend to be less often used due to an increase in systolic (3.0 mmHg) and diastolic (1.9 mmHg) blood pressure (Chapman et al., 2016).

A recent network meta-analysis (14 RCTs) compared modafinil, armodafinil, pitolisant, and solriamfetol for EDS in both CPAP-treated or untreated OSA (Pitre et al., 2023). Despite the limitations related to this kind of statistical model, the authors highlighted the greater efficacy of solriamfetol, and a higher risk of treatment discontinuation with modafinil and armodafinil at 4 weeks of treatment.

Table 1 summarizes pharmacological treatments in OSA.

**Table 1:** Pharmacological treatments in OSA.

**Drug**	**Drug class**	**Target**	**Usual effective dose**	**No. of available RCTs**	**Study duration**	**Outcome**	**Side effects**	**SE-related drug withdrawal**
Acetazolamide (Edwards et al., 2012)	Carbonic anhydrase inhibitor	LG	500 mg	13	Median 6 days	AHI reduction of 14/h	Paresthesia, dysgeusia, polyuria, fatigue	0%
Sulthiame (Hedner et al., 2022)	Carbonic anhydrase inhibitor	LG	200–400 mg	1	4 weeks	AHI decrease from 55 to 33 (400 mg) or 61 to 41 (200 mg)	Paresthesia, dyspnea	18%
Atomoxetine/oxybutynin (Taranto-Montemurro et al., 2019)	NA reuptake inhibitor + anti- muscarinic	UA muscle activity	A 80 mg O 5 mg	1	1 night	AHI decrease from 29 to 8	Urinary hesitation, dry mouth, headache	0%
Reboxetine/oxybutynin (Perger et al., 2022)	NA agent + anti- muscarinic	UA muscle activity	R 4 mg O 5 mg	1	1 week	AHI decrease from 49 to 18	Urinary hesitation, dry mouth, palpitation, insomnia	0%
Reboxetine/butylbromide (Lim et al., 2021)	NA agent + anti- muscarinic	UA muscle activity	R 4 mg B 20 mg	1	1 night	AHI decrease from 51 to 33	Urinary hesitation	8%
Benzodiazepines (Carter and Eckert, 2021) Nitrazepam Flurazepam Triazolam Temazepam	GABA-A receptor agonist	AT	N 5–10 mg F 30 mg Tr 0.25 mg Te 10 mg	5	1 night	NS on AHI reduction	Respiratory depression, sedation, muscle relaxation, poor motor coordination, dizziness, excessive next-day drowsiness	0%
Z-drugs (Carter and Eckert, 2021) Zolpidem Eszopiclone Zopiclone	GABA-A receptor agonist	AT	Zm 10–20 mg Es 3 mg Z 7.5 mg	8	1–30 nights	7 NS on AHI reduction Last one: AHI decrease from 31 to 24	Respiratory depression, sedation, poor motor coordination, dizziness and excessive next-day drowsiness	0%
Trazodone (Carter and Eckert, 2021)	Tricyclic anti-depressant	AT	100 mg	2	1 night	NS on AHI reduction	Sedation, dry mouth	0%
Pimavanserin (Messineo et al., 2022)	Anti-serotoninergic	AT	34 mg	1	1 night	NS on AHI reduction	Chest pain, arrythmias, swelling of face, eyelids, lips, tongue, throat, hands, legs, feet, or genitals	0%
Dronabinol (Carley et al., 2018)	Cannabinoid Type 1 and 2 receptor agonist	Increase of vagal afferent activity	2.5 and 10 mg	1	42 nights	AHI decrease from 26 to 19/17 (2.5 and 10 mg)	Sleepiness, drowsiness, headache, nausea, vomiting	21%
Pitolisant (Dauvilliers et al., 2020; Pépin et al., 2021)	Selective H3 receptor antagonist	Promote wakefulness	5–20 mg	2	12 weeks	ESS decrease from 15 to 9/16 to 9	Headache, insomnia	1.5–2.2%
Solriamfetol (Wang et al., 2021)	Dopamine-NA reuptake inhibitor	Promote wakefulness	75, 150, 300 mg	3	2–4 weeks	ESS decrease from −1.9 to −4.7	Headache, dry mouth, nausea, dizziness	3.4%
Modafinil (Chapman et al., 2016; Kuan et al., 2016)	Inhibit the reuptake of dopamine	Promote wakefulness	200–400 mg	10	1 d−12 weeks	ESS decrease −2.9	Headache, nausea, anxiety or nervousness, insomnia, and dizziness	12%
Armodafinil (Chapman et al., 2016; Kuan et al., 2016)	Inhibit the reuptake of dopamine	Promote wakefulness	50–250 mg	5	2–12 weeks	ESS decrease −2.8	Headache, nausea, anxiety or nervousness, insomnia, and dizziness	12%

RCT, randomized controlled trial; LG, loop gain; AT, arousal threshold; SE, side effects; NA, noradrenaline; AHI, apnea-hypopnea index; ESS, Epworth sleepiness scale; NS, not statistically significant; Nb, number.

Globally, the best AHI reduction is obtained with acetazolamide, atomoxetine/oxybutinine and reboxetine/oxybutinin. Moreover, these (combination of) agents are well-tolerated, with no side effects related drug withdrawal, but caution is called for when analyzing the results, given the small number of available studies, except for acetazolamide. Acetazolamide can induce paresthesia, dysgeusia, polyuria and fatigue while atomoxetine/oxybutinine and reboxetine/oxybutynin generally lead to headache, urinary hesitation, dry mouth, palpitation, and insomnia.

For drugs promoting wakefulness, pitolisant and solriamfetol (300 mg) achieves the best balance between ESS reduction and side effects, with only 2.2–3.4% drug withdrawal related to side effects (insomnia, headache, dry mouth, nausea and dizziness).

## 4. Discussion

To enhance personalized medicine in OSA, several prerequisites must be met before we can define a management approach based on clinical and pathophysiological phenotypes. First, the traits should be easily measured, preferably by non-invasive methods. Second, the traits should be modifiable by available and effective therapies (medications or devices) and the impact of trait modification on OSA clinical outcomes should be known in order to guide sleep physicians in treatment decision making (Owens et al., 2015). We are currently far away from these approaches in routine clinical practice, and we continue to rely on a relatively intuitive approach, based on the patient's symptoms, morphology, co-morbidities, and age, to choose a first-line treatment (e.g., CPAP, MAD, PT) for which tolerance and adherence are often still poor.

The main available treatments rely indeed on anatomical approach, whereas, in certain well-identified patients, current knowledge suggests that a pharmacological approach would be more appropriate. It remains difficult to correctly identify these phenotypes among OSA patients.

Owens et al. (2015) have performed measurements of traits during sleep for predictive modeling of non-PAP therapy responses. Acting on one single trait was only effective in 25% of patients, and the best treatment success was obtained with a combination of treatments. However, trait measurements during PSG are rather complex and not feasible in all patients and in routine practice. Indeed, UA collapsibility can be measured during a standard PSG, when subjects wear a nasal mask attached to a pneumotachometer. The Pcrit is measured by reduction of CPAP levels determining the pressure in which the pharyngeal airway occludes. A Pcrit > 2.5 cm H_2_O would reflect a high collapsibility (PALM 1), and anatomical treatment, mainly CPAP, weight loss, should be the first line treatments. These patients correspond to clinical phenotypes A and E, see Figure 1. However, the traits are defined in NREM sleep and supine position. Collapsibility of UA in this situation may not reflect the traits of the patient during the whole night of sleep. Correctly classifying the PALM parameters seems thus easy in theory, but it is not accessible in daily practice and not available in all sleep centers.

Other easier approaches are possible, for example the identification of OSA patients that have low respiratory AT based on respiratory parameters collected during a PSG (AHI, nadir oxygen saturation, and the proportion of hypopnoeas vs. apneas) (Edwards et al., 2014). These patients are likely to belong to PALM 3 (low UA collapsibility) and to clinical phenotype C, suffering from insomnia.

The use of drug-induced sleep endoscopy to predict response to MAD or PT (Bosschieter et al., 2022) is also a good option, but not yet routinely implemented.

On the other hand, data regarding pharmacological approaches in OSA are scarce and rely on short-duration studies in very small samples of patients. RCTs in this field are generally underpowered and their conclusions should be interpreted with caution.

EDS treatments are the exception, with larger series with well-documented drug effectiveness. However, pharmacological treatments are undeniably an excellent alternative to existing treatments in OSA as they are less intrusive, less constraining, and more acceptable for patients (Aurora et al., 2015).

### 4.1. How to choose medications in unselected OSA?

Some features of clinical OSA phenotypes can help clinicians to identify underlying pathophysiological traits. Even without Pcrit measurements, PSG characteristics can direct PALM classification, keeping in mind that, in the majority of patients, several PALM traits co-exist. Based on PSG data and mathematical models (Bosi et al., 2020), anatomic collapsibility is more likely if severe AHI, obstructive apneas (rather than hypopneas), and UA resistance syndrome are observed on PSG. Low AT is likely when 2/3 of PSG variables are present among: AHI <30, hypopnea/apnea ratio > 58.5%, and nadir oxygen saturation > 82.5%, or in case of UA resistance syndrome. High LG is supported by the co-occurrence of OSA and Cheynes-Stokes breathing, and a high proportion of central or mixed events, all predominant in NREM sleep.

We can suppose, based on these observations, that some clinical OSA phenotypes could be good candidates for pharmacological treatments targeting LG or AT (or residual EDS) while other phenotypes with signs of high anatomic collapsibility and high AT should be offered CPAP or UA surgery as first line treatment, see Figure 1.

Further studies testing the hypothesis that “less severe” clinical phenotypes (C and D) could benefit from pharmacological treatment should be started to confirm that these patients can benefit from alternatives to current, more “anatomical”, OSA treatments.

### 4.2. Unmet needs and future directions

Gray areas remain numerous in understanding the full picture of OSA and guiding treatment choices. There is a need to develop simplified techniques, that are widely applicable in routine clinical practice, to accurately identify the pathophysiological endotype of OSA patients, and to easily connect these with known clinical phenotypes. Moreover, the impacts of endotype and phenotype identification on treatment choice/adherence/response and patient-related outcomes (including cardiovascular, metabolic, neurocognitive comorbidities) should be further studied. New targeted therapies have been recently developed, but study durations and sample sizes have thus far been limited and generalization of the results is not yet possible. Knowledge about mid- and long-term effectiveness and tolerance, for single or combination pharmacological treatments would expand the field of personalized medicine in this very heterogeneous disorder.

## Author contributions

MB: Conceptualization, Methodology, Supervision, Validation, Writing—original draft, Writing—review and editing.

## References

[B1] American Academy of Sleep Medicine (2014). The International Classification of Sleep Disorders, 3rd Edn. Darien, IL: American Academy of Sleep Medicine.

[B2] Arzt M. Oldenburg O. Graml A. Erdmann E. Teschler H. Wegscheider K.. (2017). Phenotyping of sleep-disordered breathing in patients with chronic heart failure with reduced ejection fraction-the SchlaHF registry. J. Am. Heart Assoc. 6, e005899. 10.1161/JAHA.116.005899 29187390 PMC5778994

[B3] Aurora R. N. Collop N. A. Jacobowitz O. Thomas S. M. Quan S. F. Aronsky A. J. (2015). Quality measures for the care of adult patients with obstructive sleep apnea. J. Clin. Sleep Med. 11, 357–383. 10.5664/jcsm.4556 25700878 PMC4346655

[B4] Berry R. B. Kouchi K. Bower J. Prosise G. Light R. W. (1995). Triazolam in patients with obstructive sleep apnea. Am. J. Respir. Crit. Care Med. 151, 450–454. 10.1164/ajrccm.151.2.7842205 7842205

[B5] Bosi M. De Vito A. Eckert D. Steier J. Kotecha B. Vicini C.. (2020). Qualitative phenotyping of obstructive sleep apnea and its clinical usefulness for the sleep specialist. Int. J. Environ. Res. Public Health 17, 2058. 10.3390/ijerph17062058 32244892 PMC7143772

[B6] Bosschieter P. F. N. Vonk P. E. de Vries N. (2022). The predictive value of drug-induced sleep endoscopy for treatment success with a mandibular advancement device or positional therapy for patients with obstructive sleep apnea. Sleep Breath. 26, 1153–1160. 10.1007/s11325-021-02501-1 34596877

[B7] Carberry J. C. Fisher L. P. Grunstein R. R. Gandevia S. C. McKenzie D. K. Butler J. E.. (2017). Role of common hypnotics on the phenotypic causes of obstructive sleep apnoea: paradoxical effects of zolpidem. Eur. Respir. J. 50, 1701344. 10.1183/13993003.01344-2017 29284686

[B8] Carley D. W. Prasad B. Reid K. J. Malkani R. Attarian H. Abbott S. M.. (2018). Pharmacotherapy of apnea by cannabimimetic enhancement, the PACE clinical trial: effects of dronabinol in obstructive sleep apnea. Sleep 41, zsx184. 10.1093/sleep/zsx184 29121334 PMC5806568

[B9] Carrasco-Llatas M. O'Connor-Reina C. Calvo-Henríquez C. (2021). The role of myofunctional therapy in treating sleep-disordered breathing: a state-of-the-art review. Int. J. Environ. Res. Public Health 8, 7291. 10.3390/ijerph18147291 34299742 PMC8306407

[B10] Carter S. G. Eckert D. J. (2021). Effects of hypnotics on obstructive sleep apnea endotypes and severity: novel insights into pathophysiology and treatment. Sleep Med. Rev. 58, 101492. 10.1016/j.smrv.2021.101492 33965721

[B11] Chapman J. L. Vakulin A. Hedner J. Yee B. J. Marshall N. S. (2016). Modafinil/armodafinil in obstructive sleep apnoea: a systematic review and meta-analysis. Eur. Respir. J. 47, 1420–1428. 10.1183/13993003.01509-2015 26846828

[B12] Dauvilliers Y. Verbraecken J. Partinen M. Hedner J. Saaresranta T. Georgiev O.. (2020). Pitolisant for daytime sleepiness in patients with obstructive sleep apnea who refuse continuous positive airway pressure treatment. A randomized trial. Am. J. Respir. Crit. Care Med. 201, 1135–1145. 10.1164/rccm.201907-1284OC 31917607 PMC7193861

[B13] Dutta R. Delaney G. Toson B. Jordan A. S. White D. P. Wellman A.. (2021). A novel model to estimate key obstructive sleep apnea endotypes from standard polysomnography and clinical data and their contribution to obstructive sleep apnea severity. Ann. Am. Thorac. Soc. 18, 656–667. 10.1513/AnnalsATS.202001-064OC 33064953 PMC8008997

[B14] Eckert D. J. (2018). Phenotypic approaches to obstructive sleep apnoea - New pathways for targeted therapy. Sleep Med. Rev. 37, 45–59. 10.1016/j.smrv.2016.12.003 28110857

[B15] Edwards B. A. Eckert D. J. McSharry D. G. Sands S. A. Desai A. Kehlmann G.. (2014). Clinical predictors of the respiratory arousal threshold in patients with obstructive sleep apnea. Am. J. Respir. Crit. Care Med. 190, 1293–1300. 10.1164/rccm.201404-0718OC 25321848 PMC4315811

[B16] Edwards B. A. Sands S. A. Eckert D. J. White D. P. Butler J. P. Owens R. L.. (2012). Acetazolamide improves loop gain but not the other physiological traits causing obstructive sleep apnoea. J. Physiol. 590, 1199–1211. 10.1113/jphysiol.2011.223925 22219335 PMC3381825

[B17] Gaisl T. Haile S. R. Thiel S. Osswald M. Kohler M. (2019). Efficacy of pharmacotherapy for OSA in adults: a systematic review and network meta-analysis. Sleep Med. Rev. 46, 74–86. 10.1183/13993003.congress-2019.PA4168 31075665

[B18] Gambino F. Zammuto M. M. Virzì A. Conti G. Bonsignore M. R. (2022). Treatment options in obstructive sleep apnea. Int. Emerg. Med. 17, 971–978. 10.1007/s11739-022-02983-1 35460431 PMC9135849

[B19] Gottlieb D. J. Punjabi N. M. (2020). Diagnosis and management of obstructive sleep apnea: a review. JAMA 323, 1389–1400. 10.1001/jama.2020.3514 32286648

[B20] Hedner J. Stenlöf K. Zou D. Hoff E. Hansen C. Kuhn K.. (2022). A randomized controlled clinical trial exploring safety and tolerability of sulthiame in sleep apnea. Am. J. Respir. Crit. Care Med. 205, 1461–1469. 10.1164/rccm.202109-2043OC 35202553

[B21] Heinzer R. Vat S. Marques-Vidal P. Marti-Soler H. Andries D. Tobback N.. (2015). Prevalence of sleep-disordered breathing in the general population: the HypnoLaus study. Lancet Respir. Med. 3, 310–318. 10.1016/S2213-2600(15)00043-0 25682233 PMC4404207

[B22] Kendzerska T. Mollayeva T. Gershon A. S. Leung R. S. Hawker G. Tomlinson G. (2014). Untreated obstructive sleep apnea and the risk for serious long-term adverse outcomes: a systematic review. Sleep Med. Rev. 18, 49–59. 10.1016/j.smrv.2013.01.003 23642349

[B23] Kuan Y. C. Wu D. Huang K. W. Chi N. F. Hu C. J. Chung C. C.. (2016). Effects of modafinil and armodafinil in patients with obstructive sleep apnea: a meta-analysis of randomized controlled trials. Clin. Ther. 38, 874–888. 10.1016/j.clinthera.2016.02.004 26923035

[B24] Lam J. C. M. Lui M. M. S. Ip M. S. M. (2010). Diabetes and metabolic aspects of OSA. Sleep Apnea 189–215. 10.1183/1025448x.00024809

[B25] Light M. Owens R. L. Schmickl C. N. Malhotra A. (2019). Precision medicine for obstructive sleep apnea. Sleep Med. Clin. 14, 391–398. 10.1016/j.jsmc.2019.05.005 31375207 PMC8931687

[B26] Lim R. Messineo L. Grunstein R. R. Carberry J. C. Eckert D. J. (2021). The noradrenergic agent reboxetine plus the antimuscarinic hyoscine butylbromide reduces sleep apnoea severity: a double-blind, placebo-controlled, randomised crossover trial. J. Physiol. 599, 4183–4195. 10.1113/JP281912 34174090

[B27] Marin J. M. Carrizo S. J. Vicente E. Agusti A. G. (2005). Long-term cardiovascular outcomes in men with obstructive sleep apnoea-hypopnoea with or without treatment with continuous positive airway pressure: an observational study. Lancet 365, 1046–1053. 10.1016/S0140-6736(05)71141-7 15781100

[B28] Mehra R. Stone K. L. Varosy P. D. Hoffman A. R. Marcus G. M. Blackwell T.. (2009). Nocturnal Arrhythmias across a spectrum of obstructive and central sleep-disordered breathing in older men: outcomes of sleep disorders in older men (MrOS sleep) study. Arch. Intern. Med. 169, 1147–1155. 10.1001/archinternmed.2009.138 19546416 PMC2802061

[B29] Messineo L. Gell L. Calianese N. Sofer T. Vena D. Azarbarzin A.. (2022). Effect of pimavanserin on the respiratory arousal threshold from sleep: a randomized trial. Ann. Am. Thorac. Soc. 19, 2062–2069. 10.1513/AnnalsATS.202205-419OC 35947827 PMC9743476

[B30] Messineo L. Taranto-Montemurro L. Azarbarzin A. Oliveira Marques M. D. Calianese N. White D. P.. (2018). Breath-holding as a means to estimate the loop gain contribution to obstructive sleep apnoea. J. Physiol. 596, 4043–4056. 10.1113/JP276206 29882226 PMC6117550

[B31] O'Connor-Reina C. Rodriguez-Alcala L. Ignacio J. M. Baptista P. Garcia-Iriarte M. T. Plaza G. (2023). Assessment of muscular weakness in severe sleep apnea patients: a prospective study. Otolaryngol. Head Neck Surg. 10.1002/ohn.283 36939539

[B32] Owens R. L. Edwards B. A. Eckert D. J. Jordan A. S. Sands S. A. Malhotra A.. (2015). An integrative model of physiological traits can be used to predict obstructive sleep apnea and response to non positive airway pressure therapy. Sleep 38, 961–970. 10.5665/sleep.4750 25515107 PMC4434563

[B33] Pépin J. L. Georgiev O. Tiholov R. Attali V. Verbraecken J. Buyse B.. (2021). Pitolisant for residual excessive daytime sleepiness in OSA patients adhering to CPAP: a randomized trial. Chest 159, 1598–1609. 10.1016/j.chest.2020.09.281 33121980

[B34] Peppard P. E. Young T. Palta M. Skatrud J. (2000). Prospective study of the association between sleep-disordered breathing and hypertension. N. Engl. J. Med. 342, 1378–1384. 10.1056/NEJM200005113421901 10805822

[B35] Perger E. Taranto Montemurro L. Rosa D. Vicini S. Marconi M. Zanotti L.. (2022). Reboxetine plus oxybutynin for OSA treatment: a 1-week, randomized, placebo-controlled, double-blind crossover trial. Chest 161, 237–247. 10.1016/j.chest.2021.08.080 34543665 PMC10835052

[B36] Pevernagie D. A. Gnidovec-Strazisar B. Grote L. Heinzer R. McNicholas W. T. Penzel T.. (2020). On the rise and fall of the apnea-hypopnea index: a historical review and critical appraisal. J. Sleep Res. 29, e13066. 10.1111/jsr.13066 32406974

[B37] Pitre T. Mah J. Roberts S. Desai K. Gu Y. Ryan C.. (2023). Comparative efficacy and safety of wakefulness-promoting agents for excessive daytime sleepiness in patients with obstructive sleep apnea: a systematic review and network meta-analysis. Ann. Intern. Med. 176, 676–684. 10.7326/M22-3473 37155992

[B38] Rashid N. H. Zaghi S. Scapuccin M. Camacho M. Certal V. Capasso R. (2021). The value of oxygen desaturation index for diagnosing obstructive sleep apnea: a systematic review. Laryngoscope 131, 440–447. 10.1002/lary.28663 32333683

[B39] Schmickl C. N. Landry S. A. Orr J. E. Chin K. Murase K. Verbraecken J.. (2020). Acetazolamide for OSA and central sleep apnea: a comprehensive systematic review and meta-analysis. Chest 158, 2632–2645. 10.1016/j.chest.2020.06.078 32768459 PMC7768933

[B40] Tamura A. Kawano Y. Watanabe T. Kadota J. (2008). Relationship between the severity of obstructive sleep apnea and impaired glucose metabolism in patients with obstructive sleep apnea. Respir. Med. 102, 1412–1416. 10.1016/j.rmed.2008.04.020 18606532

[B41] Taranto-Montemurro L. Messineo L. Sands S. A. Azarbarzin A. Marques M. Edwards B. A.. (2019). The combination of atomoxetine and oxybutynin greatly reduces obstructive sleep apnea severity. A randomized, placebo-controlled, double-blind crossover trial. Am. J. Respir. Crit. Care Med. 199, 1267–1276. 10.1164/rccm.201808-1493OC 30395486 PMC6519859

[B42] Wang J. Yang S. Li X. Wang T. Xu Z. Xu X.. (2021). Efficacy and safety of solriamfetol for excessive sleepiness in narcolepsy and obstructive sleep apnea: findings from randomized controlled trials. Sleep Med. 79, 40–47. 10.1016/j.sleep.2020.12.039 33472129

[B43] Zinchuk A. Yaggi H. K. (2020). Phenotypic subtypes of OSA: a challenge and opportunity for precision medicine. Chest 157, 403–420. 10.1016/j.chest.2019.09.002 31539538 PMC7005379

[B44] Zinchuk A. V. Gentry M. J. Concato J. Yaggi H. K. (2017). Phenotypes in obstructive sleep apnea: a definition, examples and evolution of approaches. Sleep Med. Rev. 35, 113–123. 10.1016/j.smrv.2016.10.002 27815038 PMC5389934

